# Validation of the Italian version of the dissociative experience scale for adolescents and young adults

**DOI:** 10.1186/s12991-016-0120-4

**Published:** 2016-11-10

**Authors:** Concetta De Pasquale, Federica Sciacca, Zira Hichy

**Affiliations:** 1 Department of Medical Surgeon Science and Applied Technology. GF Ingrassia, University of Catania, Catania, Italy; 2 Department of Education Science, University of Catania, Via Teatro Greco, 84, Catania, Italy; 3 Department of Education of Education Science, University of Catania, Via Teatro Greco, 84, Catania, Italy

**Keywords:** A-DES, Adolescents, Young adults, Dissociative experience, Validation, Scale, Questionnaire

## Abstract

**Background:**

The Dissociative Experience Scale for adolescent (A-DES), a 30-item, multidimensional, self-administered questionnaire, was validated using a large sample of American young people sample. We reported the linguistic validation process and the metric validity of the Italian version of A-DES in the Italy.

**Methods:**

A set of questionnaires was provided to a total of 633 participants from March 2015 to April 2016. The participants consisted of 282 boys and 351 girls, and their average age was between 18 and 24 years old. The translation process consisted of two consecutive steps: forward–backward translation and acceptability testing. The psychometric testing was applied to Italian students who were recruited from the Italian Public Schools and Universities in Sicily. Informed consent was obtained from all participants at the research. All individuals completed the A-DES. Reliability and validity were tested.

**Results:**

The translated version was validated on a total of 633 Italian students. The reliability of A-DES total is .926. It is composed by 4 subscales: Dissociative amnesia, Absorption and imaginative involvement, Depersonalization and derealization, and Passive influence. The reliability of each subscale is: .756 for dissociative amnesia, .659 for absorption and imaginative involvement, .850 for depersonalization and derealization, and .743 for passive influence.

**Conclusions:**

The Italian version of the A-DES constitutes a useful instrument to measure dissociative experience in adolescents and young adults in Italy.

**Electronic supplementary material:**

The online version of this article (doi:10.1186/s12991-016-0120-4) contains supplementary material, which is available to authorized users.

## Background

Dissociation can be defined as a lack of integration of thoughts, feeling, and experiences into the normal stream of consciousness [1]. Putman has identified four categories of dissociation including memory dysfunction, disturbances in identity, passive influence, and absorption [2]. Dissociative memory dysfunctions are a form of amnesia for events, intrusive memories or flashbacks. They include phenomena such as the inability to understand if a memory is an actual event or information obtained by hearing, thinking, or reading about the event. Disturbances in identity include feelings of being more than one person (dissociative identity), distortions in the perceptions of one’s own body (depersonalization), and the inability to remember important personal information (dissociative amnesia). Passive influence involves a feeling that one’s behaviors are caused by a force from within. Absorption refers to a very intense focusing of attention [3]. Dissociative experiences can happen to everyone, but in adolescent populations, they are more common in adolescents populations than in adults population [4]. In recent decades, the progress of technology and internet has come to develop some “side effects”. Zanon et al. showed a possible correlation between the use of the internet, the dissociative experience and the presence of specific personality traits [5], while Craparo correlated internet addiction with alexithymia and dissociative experience [6].

We report the linguistic validation process and the metric validity of the Italian version of the A-DES in the Italy.

## Methods

### Sample

Participants were randomly selected from Italian Public High Schools and University in Sicily. Participants in the High Schools were selected after the informed consent granted by the head teacher, and the questionnaires were administered during regular classes. Participants from University were contacted in various study rooms. Informed consent was obtained from all participants at the research.

### General characteristics

The following parameters were collected from the students: gender, age, school or university, main use of smartphones.

#### Dissociative experience scale

The A-DES is a well-validated specific questionnaire for adolescents with dissociative symptoms in USA [1] that includes 30 questions describing memory dysfunctions, disturbances in identity, passive influence, and absorption [2]. Each item presents a statement in the first person form (e.g., “My body feels as if it doesn’t belong to me”). Under each of these statements, subjects mark the frequency of these experiences on a scale from 0 to 10 with 0 labeled “never” and 10 labeled “always”. The Total of A-DES scores are equal to the mean of all item scores. Subscale scores can also be calculated in four areas: dissociative amnesia (items 2, 5, 8, 12, 15, 22 and 27), absorption and imaginative involvement (items 1, 7, 10, 18, 24 and 28), depersonalization and derealization (items 3, 6, 9, 11, 13, 17, 20, 21, 25, 26, 29, and 30), and passive influence (items 4, 14, 6, 9 and 23) [1].

### General organization

The development and linguistic validation of a questionnaire is based on two main steps. They were organized as follows: 1. the translation and the cultural adaptation process 2. The psychometric testing. The two steps were planned under the coordination of a team that included the Italian Researcher, Psychologist and Psychiatrist to of the University of Catania.

#### Translation and cultural adaptation process

The developers provided a conceptual definition of the original items to clarify the notions investigated in each item of the original American questionnaire. The original version of the scale was drawn by five American psychologist of “The University of Arkansas” [1]. The translation and the cultural adaptation processes were organized into steps. Forward translation of the SAS-SV from English into Italian was performed by two native Italian speakers who were also fluent in English. Any differences between the 2 translated versions were discussed by the translators. An agreed upon (?) forward-translated version of the A-DES was produced. The misinterpretation and acceptability were checked. Some terms were reworded and a new version was produced.

#### Psychometric testing

The latest version was validated in a larger sample of Italian students to test the psychometric properties and to check the reliability.

### Statistical analyses

A confirmatory factor analysis was performed using the SPSS 22 software (Statistical Package for Social Science).

The reliability of instruments were calculated using Cronbach’s alpha.

## Results

### Sample characteristics

The study sample included 633 (*M* = 317.25, SD = 183.16) Italian student recruited from Public Schools and Universities and selected from March 2015 to April 2016. The participants consisted of 282 boys and 351 girls, aged between 13 and 24 years old (*M* = 18.24, SD = 3.05). Table 1 reports the distribution of the socio-demographic variables of the sample.

**Table 1:** Distribution of the socio-demographic variables of the sample

Variables	N	(N=633)	Mean
		%	
Age	633		18.24
Sex			
Males	282	44.5	
Females	351	55.5	
School/University			
Art school	1	.2	
Catering collage	93	13.6	
Archaeology faculty	1	.2	
Architecture faculty	10	1.6	
Economic faculty	3	.5	
Pharmacy faculty	1	.2	
Jurisprudence faculty	7	1.1	
Engineering faculty	21	3.3	
Medical faculty	3	.5	
Psychology faculty	146	23.1	
Education faculty	111	17.5	
Secondary school specializing in scientific subjects	200	31.6	
Tourism faculty	2	.3	
Physical education	4	.6	
Natural science	1	.2	
Social science	4	.6	
Tourism high school	25	4.0	

*N* sample number,  *%* percentage

### Item analysis and reliability

Table 2 shows item analysis of four factor of Dissociative Experience Scale for Adolescent for Italian samples. Regarding internal consistency all the item-total correlations appeared adequate, and there were no changes in the value of alpha excluding item. Finally In the end all alpha coefficients (Dissociative Amnesia = .76, Absorption and imaginative involvement = .66; Depersonalization and derealization = .85; Passive influence = .74) and the split-half correlation (Dissociative Amnesia = .63, Absorption and imaginative involvement = .48; Depersonalization and derealization = .67; Passive influence = .57) were adequate.

**Table 2:** Item analysis of four factor of dissociative experience scale for adolescent for Italian samples

	M	SD	Item-total correlation	Alpha if item deleted	Factorloading
Dissociative amnesia					
2	1.32	2.178	.433	.925	.48
5	2.60	2.946	.538	.923	.57
8	1.29	2.370	.619	.922	.68
12	2.74	2.688	.408	.925	.41
15	1.18	2.347	.522	.923	.57
22	1.10	2.248	.573	.923	.63
27	2.29	3.070	.599	.922	.61
Absorptionand imaginative involvement					
1	2.85	2.549	.280	.927	.32
7	2.60	2.579	.85	.924	.51
10	4.54	3.027	.347	.926	.34
18	2.52	2.862	.570	.923	.66
24	1.40	2.402	.592	.923	.69
28	1.07	2.175	.529	.923	.54
Depersonalization andderealization					
3	2.49	2.579	.449	.924	.45
6	1.11	2.127	.637	.922	.63
9	1.85	2.636	.415	.925	.42
11	2.64	3.124	.501	.924	.49
13	.82	2.030	.563	.923	.66
17	3.97	3.224	.321	.927	.35
20	2.35	2.951	.604	.922	.63
21	1.61	2.611	.595	.922	.67
25	1.30	2.504	.608	.922	.70
26	1.94	2.797	.625	.922	.68
29	1.21	2.520	.566	.923	.64
30	.75	1.995	.642	.922	.75
Passive influence					
4	3.27	2.794	.472	.924	.49
14	2.12	2.809	.568	.923	.58
16	1.47	2.302	.581	.923	.63
19	2.78	3.000	.588	.922	.64
23	1.41	2.450	.661	.922	.72

*M* mean, *SD* standard deviation

### Factor structure

Because the relationship between observed variables and their underlying latent constructs was already confirmed in previous study [1], in this study we performed a confirmatory factor analysis [7] to verify if the same relationships can be find in our sample. To verify the adequacy of the models we used the χ^2^: a solution fits well the data well when χ^2^ is non-significant (*p* > .05). Given that this statistic is sensitive to sample size, the two-index strategy [8] proposing combined use of comparative fit index [9] and standardized root mean square residual [10] was applied. The model fits the data well if CFI is greater than or equal to .95 and SRMR is smaller than or equal to .08. Goodness of fit indexes are: χ^2^(399) = 2132.88, *p* < .001, CFI = .96, SRMR = .067; as you can see, although the χ^2^ is significant, SRMR and CFI meet completely the criteria. Moreover, all factor loadings were significant, *p* < .001 (Table 2).

We also tested a second order factor structure whit one superordinate factor [7]. Goodness of fi indexes are: χ^2^(401) = 2163.40, *p* < .001, CFI = .96, SRMR = .067; also in this case, although the χ^2^ is significant, SRMR and CFI meet completely the criteria. Moreover, all factor loadings were significant, *p* < .001 (Table 3). Finally, all inter-factor correlations were significant (p < .001) and reliability coefficient were high (alpha = .93; split-half correlation = .76).

**Table 3:** Factor structure of DES

		M	SD	Factorloading	Correlation			
					1	2	3	4
1	Amnesia	1.7883	1.63723	.96				
2	Absorption	2.4953	1.58881	.94	.721			
3	Depersonalization	1.8372	1.60949	.91	.734	.664		
4	Influence	2.2079	1.88347	.98	.716	.653	.753	
DES_Tot		2.0192	1.47459					

*M* mean, *SD* standard deviation

## Discussion

The scale possessed a good internal consistency, all factors show good saturation. The DES in Italian version is valid. This scale is useful for detecting the dissociative experience and various events such as amnesia, depersonalization, passive influence and absorption.

It can be used to implement prevention projects in the screening.

The scale can be used both as a single factor, both with the four separate factors (Dissociative Amnesia, Absorption and imaginative involvement, Depersonalization and derealization, and Passive influence).

We reporte in Additional file 1 Italian version of the dissociative experience scale for Adolescent.

## Conclusion

New technologies related to social communication have made problematic the quality of existence. The Teenagers now spends more and more time in front of the smartphone and the Internet, mainly to communicate with others through messages, social networks, calls, finding in them a more accessible means of communication more accessible, easy, free from anxiety and fear, a defense from on the other. It brings more and more to escape from the real relationship.

Literature shows they are absent for other dissociative disorders such as significant “dissociative trance from display screen” shown in the study of Caretti and coworkers [11].

Directly proportional to the degree of reliance on smartphones is the presence of mild dissociative symptoms related to the size of the ‘‘ absorption and imaginative assimilation”, the tendency to engage his their mind in situations of altered and highly focused attention [12].

The aim of this study was to test the validity of the Dissociative Experience scale in Italian to analyze dissociative experience in Italian adolescents who use technological communication instruments.

At present there is insufficient evidence-based literature to establish diagnostic criteria and clinical symptoms needed to identify repetitive patterns of behavior and excessive, comparable to those produced by “Disorders related to substances and disorders Addiction” [13]. The use of this instrument can be an added value for a useful comparison on important issues that limit the educational process and human planning, preventing him from seizing the opportunities necessary for it to achieve maturation and personality development in all his physical and psychic potential, intellectual and moral.

## Additional file

**Additional file 1: Appendix.** Italian version of the dissociative experience scale for Adolescent.
