# Supplementary material for: Validation of the Italian version of the dissociative experience scale for adolescents and young adults
Source: Ann Gen Psychiatry. 2016 Nov 10;15:31. doi: 10.1186/s12991-016-0120-4 (PMC5103436; doi:10.1186/s12991-016-0120-4)
Supplement: Supplementary file 1 — Additional file 1: Appendix. Italian version of the dissociative experience scale for Adolescent. [file 12991_2016_120_MOESM1_ESM.docx]

**Appendix**

**Italian version of the Dissociative Experience Scale for Adolescent**

| - Sono così coinvolto a guardare la TV, leggere, o giocare ad un video gioco che non ho alcuna idea di cosa sta succedendo intorno a me. |
| --- |
| - Mi ritrovo compiti in classe o compiti a casa che non ricordo di aver fatto. |
| - Ho forti sentimenti che non sembrano essere miei. |
| - So fare qualcosa veramente bene una volta e altre volte no. |
| - Le persone mi dicono che faccio o dico cose che non ricordo di aver detto o di aver fatto. |
| - Mi sento come se fossi in una nebbia o sballato e le cose intorno a me sembrano irreali. |
| - Mi sento confuso a stabilire se ho fatto qualcosa o ho solo pensato di farla. |
| - Guardo l’orologio e mi rendo conto che il tempo è trascorso e non riesco a ricordare cosa è successo. |
| - Sento voci nella mia testa che non sono mie. |
| - Quando sono in qualche posto in cui non voglio essere so andar via con la mente. |
| - Sono cosi bravo a mentire e recitare che io stesso mi credo. |
| - Mi sorprendo a “svegliarmi” mentre sto facendo qualcosa. |
| - Non mi riconosco allo specchio. |
| - Mi ritrovo ad andare da qualche parte o fare qualcosa e non so perché. |
| - Mi ritrovo da qualche parte e non ricordo come ci sono arrivato. |
| - Ho pensieri che non sembrano appartenermi veramente. |
| - Trovo che posso superare il dolore fisico. |
| - Non riesco a capire se le cose sono realmente successe o se ho solo sognato o pensato che lo siano. |
| - Mi ritrovo a fare qualcosa che so che è sbagliata, anche quando non voglio realmente farla. |
| - Le persone mi dicono che a volte mi comporto così differentemente dal solito che sembro essere una persona diversa. |
| - Sembra che ci siano delle barriere dentro la mie mente. |
| - Trovo scritti, disegni o lettere che devo aver fatto ma che non ricordo . |
| - Qualcosa dentro di me sembra farmi fare cose che non voglio fare. |
| - Mi capita di non riuscire a dire se sto solo ricordando qualcosa o se essa mi sta realmente accadendo. |
| - Mi ritrovo a stare al difuori del mio corpo, a guardarmi come se fossi un’altra persona. |
| - Le mie relazioni con la mia famiglia e i miei amici cambiano improvvisamente e non so perché. |
| - Sento come se il mio passato sia un puzzle ed alcuni pezzi sono mancanti. |
| - Sono così coinvolto dai miei giochi che sembranoreali. |
| - Mi sento come se ci fossero diverse persone dentro di me. |
| - Il mio corpo sembra non appartenermi. |
